# Annual operational data for the 10 MWe ELLO Fresnel concentrated solar power plant

**DOI:** 10.1016/j.dib.2026.113278

**Published:** 2026-09-18

**Authors:** Valentine Grange, Fabien Roget, Valéry Vuillerme, Quentin Falcoz, Sylvain Rodat

**Affiliations:** a Processes, Materials and Solar Energy Laboratory, PROMES-CNRS, 66120 Font-Romeu, France; b eLLO solar power plant, 66800 Llo, France; c Univ. Grenoble Alpes, CEA, LITEN, DTCH, L2TS, 38000, Grenoble, France

**Keywords:** Concentration solar technology (CST), Linear fresnel reflector (LFR), Standard daily operations, Thermal energy storage (TES), Direct steam generation (DSG)

## Abstract

This data article introduces a unique yearly dataset from the 10 MWe eLLO Concentrated Solar Power (CSP) plant, a commercial-scale Linear Fresnel Reflector (LFR) facility using Direct Steam Generation (DSG) and steam storage, located in Llo, France. The dataset, collected via a supervisory system, includes real-time operational data from 124 sensors distributed across the plant, categorized into seven types: meteorological, solar field (east/west), turbine/aero-condenser, feedwater, electricity production and storage system. Those data were collected under standard operational conditions at two different acquisition times:

• a 24-hour snapshot (from July 16th, 2025, 04:00 to July 17th, 2025, 04:00) recorded with a 10-second interval.
• a year-long variable data (from January 1st, 2025, 00:00 to January 1st, 2026, 00:00) with a 1-hour interval. Erroneous data were flagged as NaN, resulting in 338 usable days for 2025.

The dataset is organized into three sheets:

• “Description”: metadata (sensors IDs, variables, units, annotated schematics).
• “24-hour period”: data for all variables over the 24-hour period described earlier.
• “Year 2025″: data for all variables over the year 2025.

This is the first open yearly dataset for a commercial LFR-DSG plant, enabling experimental validation of numerical models, simulations of part-load operation, thermal storage dynamics, and efficiency, as well as optimization of plant sizing, operational strategies, and techno-economic analyses. A reference case study for researchers, engineers and industrial stakeholders in a field where real-world data are scarce.

Specifications Table

**Table:** 

Subject	Engineering & Materials science
Specific subject area	This dataset concerns Concentrated Solar Power (CSP) and Concentrated Solar Thermal (CST) plants, particularly those using Linear Fresnel Reflector (LFR) technology and Direct Steam Generation (DSG).
Type of data	Table (.csv,.xlsx), Filtered data Flow diagrams
Data collection	The dataset comprises experimental on-site measurements collected at the eLLO concentrated solar power plant. The data were acquired through the plant’s sensor network during normal daily operation and thus reflect the real operating conditions of an LFR-DSG power plant.
Data source location	• Institution: eLLO concentrated solar power plant • City/Town/Region: Llo/Occitanie • Country: France • Latitude and longitude: 42.466798, 2.071873
Data accessibility	Repository name: Annual operational data for the 10 MWe ELLO Fresnel concentrated solar power plant Data identification number: https://doi.org/10.57745/4MWMMQ Direct URL to data: https://entrepot.recherche.data.gouv.fr/privateurl.xhtml?token=7156d470–2650–4242-af00-d59716daa8a7
Related research article	None

## 1 Value of the Data

• A representative yearly dataset, capturing the nominal operation of a Linear Fresnel Reflector (LFR) plant using Direct Steam Generation (DSG) is described.
• These data are valuable as they enable performance assessment of a CSP/CST plant using LFR technology and direct steam generation, including part-load operation, by providing key variables such as Direct Normal Irradiance (DNI), mass flow rates, temperatures and pressures.
• The dataset can be used to develop and validate 0D, 1D, and CFD models, as well as to design new optimization tools for an LFR - DSG plant.
• This dataset is valuable for both researchers and industrial stakeholders involved in renewable energy development, as CST / CSP plants can substitute fossil fuels for both heat [1] and electricity [2] generation.
• The data support optimized plant sizing, realistic techno-economic analyses, and the integration of this technology with industrial processes.

## 2 Background

Concentrated Solar Technologies (CST) are critical for advancing renewable energy solutions. However, a significant gap exists in the availability of comprehensive operational datasets for these systems. Existing datasets present notable limitations. For instance, Sergio et al [3] provide meteorological data and images only, while Soto et al [4] covers a few days of operational data at most. Kraft et al [5], though potentially comprehensive, is not publicly accessible due to confidentially restrictions. Some are limited to prototypes [6], partial measurements [7], or single-field studies (such as optics [8]). Consequently, these datasets primarily address simulations, short-term experiments, or specific technical aspects, rather than full-scale, long-term operational performance.

To address this gap, a comprehensive operational dataset was recovered from the eLLO plant, a commercial, first-of-a-kind Linear Fresnel Reflector (LFR) - Direct Steam Generation (DSG) plant. The dataset was collected under normal operational conditions, using standard monitoring and control systems. The plant features a unique 4-hour steam storage system.

Following COP30 in Brazil, which highlighted the urge to accelerate industrial decarbonization and renewable energy deployment [9], this dataset supports CST system design, performance assessment, and scalability studies. It enables reproducible models, techno-economic analyses, and optimization tools for new CSP/CST projects.

## 3 Data Description

### 3.1 Dataset

The dataset is organized into three sheets, available in both excel (.xlsx) and CSV (.csv) formats:

• Sheet 1, named “Description”, serves as a comprehensive inventory of all sensors, including their identifier numbers, types, measured variables, and units, along with annotated schematics of the plant. Each physical sensor’s location is pinpointed to contextualize measurements across the meteorological stations, solar fields, power block, storage system and feedwater system.
• Sheet 2, named “24-hour period”, offers high-resolution temporal data, capturing all described variables at 10-seconds intervals (averaged from 1-second samples), over a 24-hour period, from July 16, 2025, 04:00, to July 17, 2025, 04:00. This acquisition period was selected to represent a best-case scenario, with high Direct Normal Irradiance (DNI) and no cloud cover, ensuring optimal conditions for plant operations. This timeframe captures the full operational cycle of the plant, including its thermal storage dispatch capabilities, covering both daytime normal operations and extended overnight production, during which stored energy is used to produce electricity.
• Sheet 3, named “year 2025″, provides the described data for the entire year 2025 with a 1-hour time step, representing averaged values over each hour: from January 1, 2025, 00:00 to January 1, 2026, 00:00. In this dataset, missing data, such as gaps resulting from scheduled maintenance, or computational server freezes, are systematically flagged with NaN values.

Additionally, annotated plant schematics are provided to offer a detailed visual representation of the facility. These schematics map the exact locations of all sensors across the three primary operational zones:

• ``Solar field'': Covering both the East (L1–L10) and West (L11–L27) solar fields.
• ``Storage system'': Highlighting sensor positions related to thermal storage.
• ``Power block'': Showing sensor placements in the turbine, condenser, feedwater, and auxiliary systems.

The dataset follows the Central European Time (CET/CEST) convention:

• UTC+01:00 (CET) from January 1 to March 30, 2025, 01:00 UTC,
• UTC+02:00 (CEST) from March 30,2025, 01:00 UTC to October 26, 2025, 01:00 UTC,
• UTC+01:00 (CET) from October 26, 2025, 01:00 UTC, to December 31

The data provided in this paper are categorized into seven zones (meteorological, West field, East field, turbine/ aero-condenser, feedwater, electricity production, and storage system), each corresponding to a specific segment of the plant. Below is a detailed description of the data for each zone, including measured variables, units and exact location (refer to sheet “Description” for schematics or to the schematics provided after the tables). The location of sensors in the solar fields may correspond to a specific solar line. Lines are numbered as follows: in the East Solar Field, there are 10 lines (L1 to L10), and in the West Solar Field, there are 17 lines (L11 to L27).

A few key points should be noted regarding the measurements. Wind direction is defined such that 0° corresponds to a wind blowing from the south to the north, and 90° to a wind blowing from the west to the east. For tank level measurements, the 0 mm reference point is set at the center of the tank, which explains why negative values may occur when the water level falls below this midpoint. Tank dimensions are detailed in Table 1. Finally, some flow rate measurements were manually corrected based on valve position (these data are not provided in the dataset): whenever the corresponding valve position was zero or negative, the flow rate was set to zero. The following flow rates were adjusted based on this principle:

• West-field tank purge flow rate [t/h].
• East-field tank purge flow rate [t/h].
• Steam flow rate at turbine 1 inlet downstream of superheater [t/h].
• Steam bypass flow rate from turbine to aero-condenser [t/h].
• Condensate flow rate from feedwater heater to deaerator [t/h].
• Demineralized water flow rate to deaerator [t/h].
• Feedwater flow rate to storage tanks [t/h].
• Steam injection flow rate to storage [t/h].
• Steam pressurization flow rate to storage [t/h].
• Storage water flow rate to West-field recirculation [t/h].

**Table 1:** Dimensions of tanks with level measurement.

	Tank	Internal height (mm)	Total volume ($m^{3}$)
**Horizontal tank**	West separator tank	2880	95,6
	East separator tank	2880	40,8
	Condensate tank	1880	12,45
	Deaerator	2500	33,7
	Storage tank (all 9 are identical)	2890	120
**Vertical tank**	Flash tank	2160	Around 2

These flowrates were further adjusted based on both the main valve position and bypass valve position:

• West-field feedwater flow rate [t/h].
• East-field feedwater flow rate [t/h].
• Steam flow rate from HP collector to superheater [t/h].
• Feedwater flow rate to East Field [t/h].
• Feedwater flow rate to West Field [t/h].

**a. Meteorological Data.**

**Table:** 

N°	Variable	Unit	Location
1	GHI = Global Horizontal Irradiance (output L11)	[W/m²]	Output L11
2	East-field DNI = Direct Normal Irradiance (Aero-condenser)	[W/m²]	Aero-condenser
3	West-field DNI = Direct Normal Irradiance (output L11)	[W/m²]	Output L11
4	West-field DNI 2= Direct Normal Irradiance (input L27)	[W/m²]	Input L27
5	External Temperature	[ °C]	Weather station, input L11
6	External Temperature, West-field low	[ °C]	West-field, input L27
7	External Temperature, West separator tank	[ °C]	West separator tank
8	External Temperature, East-field low	[ °C]	East-field, input L10
9	External Temperature, ACC zone	[ °C]	Aero-condenser
10	External Temperature, between ACC 1&2	[ °C]	Under Aero-condenser
11	External Temperature, between ACC 2&3	[ °C]	Under Aero-condenser
12	Wind Speed at 1 m height	[km/h]	Weather station, L11
13	Wind Direction at 1 m height	[°]	Weather station, L11
14	Wind Speed at 10 m height	[km/h]	Weather station, L11
15	Wind Direction at 10 m height	[°]	Weather station, L11
16	Atmospheric pressure in the turbine hall	[mbarA]	Turbine hall

**b. West Field Data.**

**Table:** 

N°	Variable	Unit	Location
17	Number of heliostats focusing, West Field	/	West-field
18	West-field separator tank pressure	[barg]	West-field separator tank
19	West-field separator tank level	[mm]	West-field separator tank
20	West-field feedwater flow rate	[t/h]	West-field
21	West-field steam flow rate at tank outlet	[t/h]	West-field separator tank
22	West-field tank steam dome temperature	[ °C]	West-field separator tank
23	West-field recirculation flow rate at pump discharge	[t/h]	West-field recirculation pump
24	West-field recirculation pump discharge water temperature	[ °C]	West-field recirculation pump
25	West-field recirculation pump 1 discharge pressure	[barg]	West-field recirculation pump
26	West-field recirculation pump 2 discharge pressure	[barg]	West-field recirculation pump
27	West-field common outlet temperature (lines 11 to 27)	[ °C]	West-field, L11 to L27
28	West-field tank purge flow rate	[t/h]	West-field separator tank

**c. East Field Data.**

**Table:** 

N°	Variable	Unit	Location
29	Number of heliostats focusing, East Field	/	East-field
30	East-field separator tank pressure	[barg]	East-field separator tank
31	East-field separator tank level	[mm]	East-field separator tank
32	East-field feedwater flow rate	[t/h]	East-field
33	East-field steam flow rate at tank outlet	[t/h]	East-field separator tank
34	East-field tank steam dome temperature	[ °C]	East-field separator tank
35	East-field recirculation flow rate at pump discharge	[t/h]	East-field recirculation pump
36	East-field recirculation pump discharge water temperature	[ °C]	East-field recirculation pump
37	East-field recirculation pump discharge pressure	[barg]	East-field recirculation pump
38	East-field common outlet temperature (lines 1 to 3)	[ °C]	East-field, L1 to L3
39	East-field common outlet temperature (lines 4 to 10)	[ °C]	East-field, L4 to L10
40	East-field tank purge flow rate	[t/h]	East-field separator tank

*Note:* The East-field DNI sensor (item 2) is located at the Aero-condenser, adjacent to the East solar field, for practical reasons.

**d. Turbine and Aero-condenser Data.**

**Table:** 

N°	Variable	Unit	Location
41	HP collector pressure	[barg]	High pressure collector (HP collector)
42	HP collector calculated saturation temperature	[ °C]	HP collector
43	HP collector measured temperature	[ °C]	HP collector
44	Steam flow rate to turbine	[t/h]	Flow from HP collector to superheater and resuperheater
45	Steam pressure to turbine upstream of superheater after expansion	[barg]	Flow from HP collector to superheater (after expansion)
46	Steam temperature to turbine upstream of superheater after expansion	[ °C]	Flow from HP collector to superheater (after expansion)
47	Steam pressure at turbine 1 inlet downstream of superheater	[barg]	Turbine inlet 1 (downstream of superheater)
48	Steam temperature at turbine 1 inlet downstream of superheater	[ °C]	Turbine inlet 1 (downstream of superheater)
49	Steam flow rate at turbine 1 inlet downstream of superheater	[t/h]	Turbine inlet 1 (downstream of superheater)
50	Steam flow rate from HP collector to superheater	[t/h]	Flow from HP collector to superheater
51	Steam bypass flow rate from turbine to aero-condenser	[t/h]	Bypass flow, from HP collector to ACC
52	R1 extraction pressure	[barg]	Turbine extraction 1
53	R1 extraction temperature	[ °C]	Turbine extraction 1
54	R1 extraction flow rate	[t/h]	Turbine extraction 1
55	Steam pressure to turbine upstream of resuperheater	[barg]	Flow from turbine’s first stage to resuperheater
56	Steam temperature to turbine upstream of resuperheater	[ °C]	Flow from turbine’s first stage to resuperheater
57	Steam pressure at turbine 2 inlet downstream of resuperheater	[barg]	Flow from resuperheater to turbine’s second stage
58	Steam temperature at turbine 2 inlet downstream of resuperheater	[ °C]	Flow from resuperheater to turbine’s second stage
59	Steam flow rate at turbine 2 inlet downstream of reheater	[t/h]	Flow from resuperheater to turbine’s second stage
60	Steam flow rate from HP collector to reheater	[t/h]	Flow from HP collector to resuperheater
61	Superheater and reheater flash tank pressure	[barg]	Flash tank
62	Superheater and reheater flash tank level	[mm]	Flash tank
63	R1 extraction steam pressure to feedwater heater	[barg]	Turbine extraction 1
64	R1 extraction steam temperature to feedwater heater	[ °C]	Turbine extraction 1
65	Condensate flow rate from feedwater heater to deaerator	[t/h]	Flow from feedwater heater to deaerator
66	R2 extraction pressure	[barg]	Turbine extraction 2
67	R2 extraction temperature	[ °C]	Turbine extraction 2
68	R2 extraction flow rate to deaerator	[t/h]	Turbine extraction 2
69	R2 extraction steam pressure to deaerator	[barg]	Turbine extraction 2
70	R2 extraction steam temperature to deaerator	[ °C]	Turbine extraction 2
71	R3 extraction temperature to condensate heater	[ °C]	Turbine extraction 3
72	R3 extraction flow rate to condensate heater	[t/h]	Condensate heater
73	Condensate temperature from condensate heater	[ °C]	Condensate heater
74	Turbine exhaust pressure	[barA]	Turbine exhaust
75	Turbine exhaust steam temperature	[ °C]	Turbine exhaust
76	Steam flow rate at turbine outlet	[t/h]	Turbine exhaust
77	Steam temperature at aero-condenser inlet	[ °C]	Aero-condenser inlet
78	Condensate temperature at aero-condenser 1 outlet	[ °C]	Aero-condenser outlet 1
79	Condensate temperature at aero-condenser 2 outlet	[ °C]	Aero-condenser outlet 2

**e. Return of condensates and feedwater Data.**

**Table:** 

N°	Variable	Unit	Location
80	Condensate tank level	[mm]	Condensate tank
81	Condensate tank water temperature	[ °C]	Condensate tank
82	Condensate pump discharge pressure	[barg]	Condensate pump
83	Condensate temperature at pump discharge	[ °C]	Condensate pump
84	Condensate temperature downstream of turbine steam condenser	[ °C]	Flow from condensate pump to deaerator condenser.
85	Condensate temperature downstream of deaerator steam condenser	[ °C]	Downstream of deaerator condensate
86	Condensate temperature downstream of condensate heater	[ °C]	Flow from condensate heater to deaerator
87	Condensate flow rate to deaerator	[t/h]	Flow from condensate heater to deaerator
88	Demineralized water flow rate to deaerator	[t/h]	Flow from demineralized water tank to deaerator
89	Deaerator level	[mm]	Deaerator
90	Deaerator water temperature	[ °C]	Deaerator
91	Deaerator pressure	[barg]	Deaerator
92	Feedwater pump discharge pressure	[barg]	Feedwater pump
93	Total feedwater flow rate	[t/h]	Feedwater pump
94	Feedwater temperature downstream of feedwater heater	[ °C]	Downstream of feed water heater
95	Feedwater flow rate to East Field	[t/h]	Flow from feedwater heater to East field
96	Feedwater flow rate to West Field	[t/h]	Flow from feedwater heater to West field
97	Feedwater flow rate to storage tank	[t/h]	Flow from feedwater heater to storage system

**f. Electricity production Data.**

**Table:** 

N°	Variable	Unit	Location
98	Electrical power output from generator	[MW]	Generator output
99	Electrical power at export meter	[MW]	Export meter
100	Total active electrical power at delivery substation	[kW]	Delivery substation (Grid convention: positive for plant imports, negative for exports.)

*Note:* the generator output (item 98) and export meter (item 99) are provided in megawatts (MW), while the delivery substation power (item 100) is provided in kilowatts (kW). This reflects the original monitoring configurations of the eLLO plant.

**g. Storage system Data.**

**Table:** 

N°	Variable	Unit	Location
101	Steam injection flow rate to storage	[t/h]	Steam flow from West separator tank to storage (storage)
102	Steam storage discharge flow rate	[t/h]	Flow from storage to HP collector (destorage)
103	Steam pressurization flow rate to storage	[t/h]	Flow from West separator tank to storage (pressurization)
104	Storage pump 1 suction water temperature	[ °C]	Storage pump 1
105	Storage pump 2 suction water temperature	[ °C]	Storage pump 2
106	Storage water flow rate to West-field recirculation	[t/h]	Flow from storage pump to West-field
107	Storage steam tank 1 pressure	[barg]	Steam tank 1
108	Storage steam tank 1 level	[mm]	Steam tank 1
109	Storage steam tank 2 pressure	[barg]	Steam tank 2
110	Storage steam tank 2 level	[mm]	Steam tank 2
111	Storage steam tank 3 pressure	[barg]	Steam tank 3
112	Storage steam tank 3 level	[mm]	Steam tank 3
113	Storage steam tank 4 pressure	[barg]	Steam tank 4
114	Storage steam tank 4 level	[mm]	Steam tank 4
115	Storage steam tank 5 pressure	[barg]	Steam tank 5
116	Storage steam tank 5 level	[mm]	Steam tank 5
117	Storage steam tank 6 pressure	[barg]	Steam tank 6
118	Storage steam tank 6 level	[mm]	Steam tank 6
119	Storage steam tank 7 pressure	[barg]	Steam tank 7
120	Storage steam tank 7 level	[mm]	Steam tank 7
121	Storage steam tank 8 pressure	[barg]	Steam tank 8
122	Storage steam tank 8 level	[mm]	Steam tank 8
123	Storage steam tank 9 pressure	[barg]	Steam tank 9
124	Storage steam tank 9 level	[mm]	Steam tank 9

### 3.2 Metadata

An extensive sensor layout map is provided in Sheet 1 (``Description''), illustrating the spatial distribution of all measured variables through detailed schematics of the solar field (see Fig. 1), power block (see Fig. 2), and storage system (see Fig. 3).

**Fig. 1:**
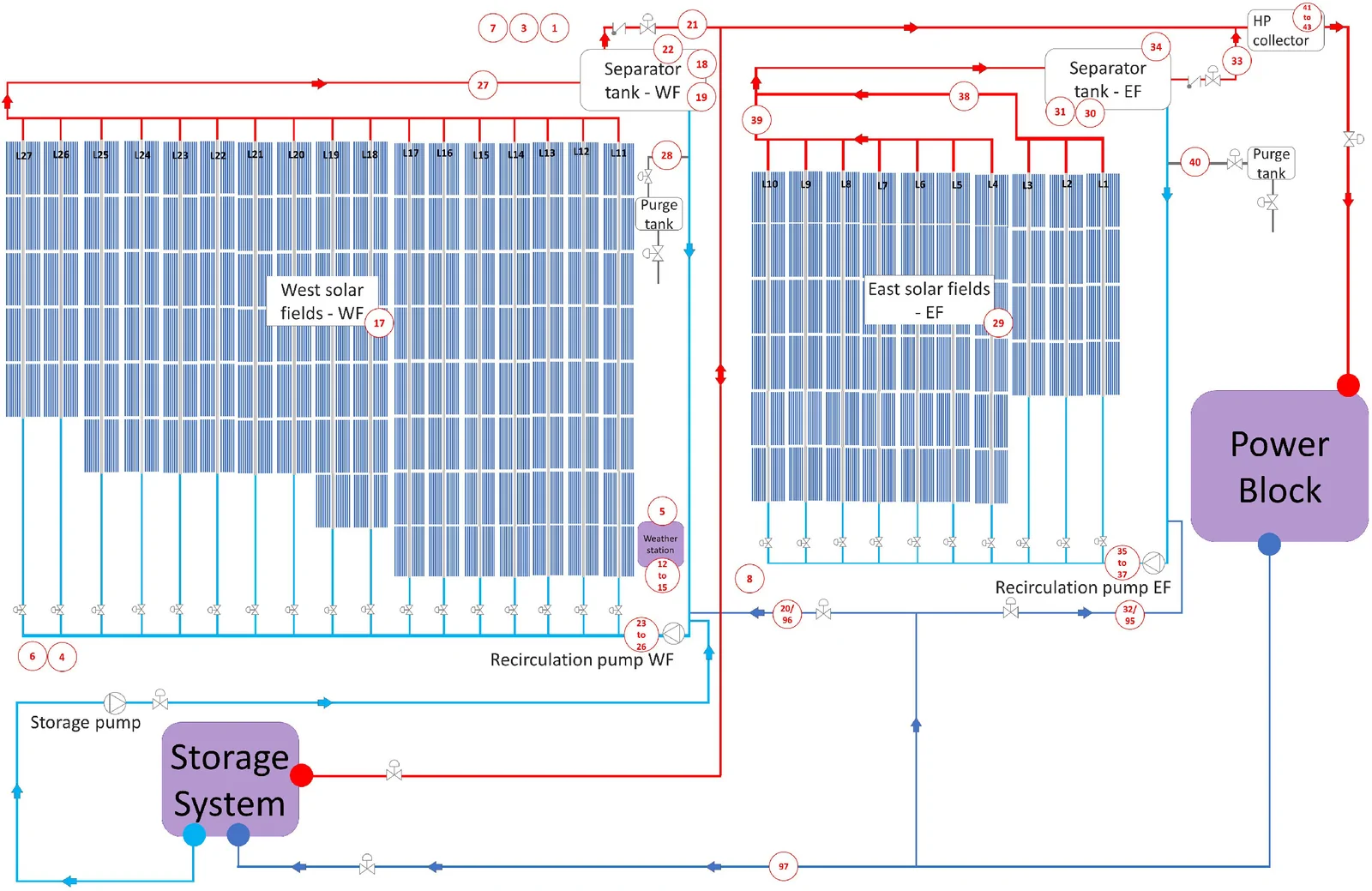
Schematic of the ELLO solar field instrumentation. Simplified flow diagram of the ELLO solar field, showing the layout of solar collector system and the positions of key sensors (e.g., temperature, flow rate, and solar radiation measurements).

**Fig. 2:**
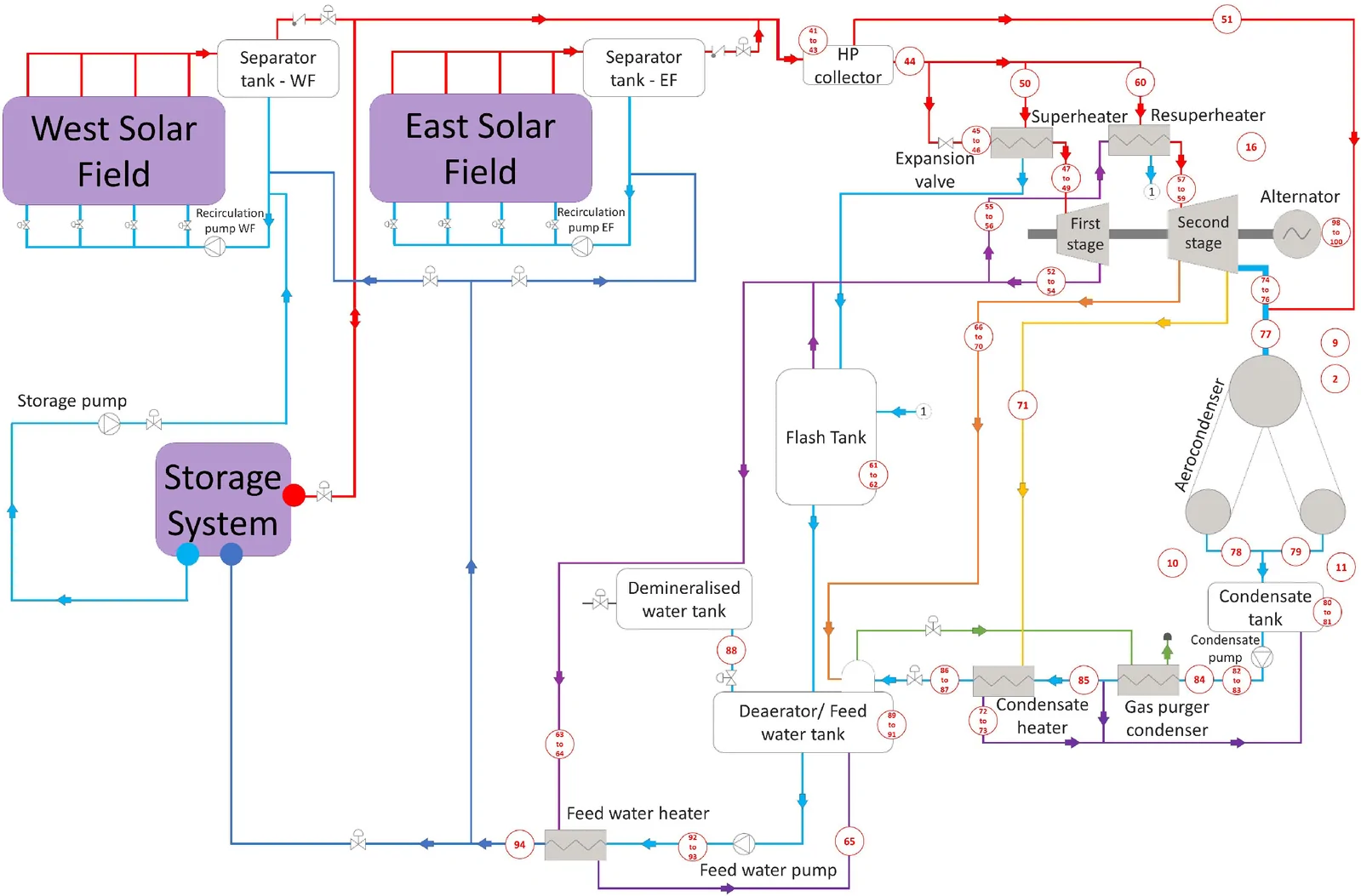
Schematic of the power block instrumentation. Simplified flow diagram of the power block, illustrating the arrangement of the aero-condenser, turbine, and associated systems, with marked positions of pressure, temperature, and flow sensors.

**Fig. 3:**
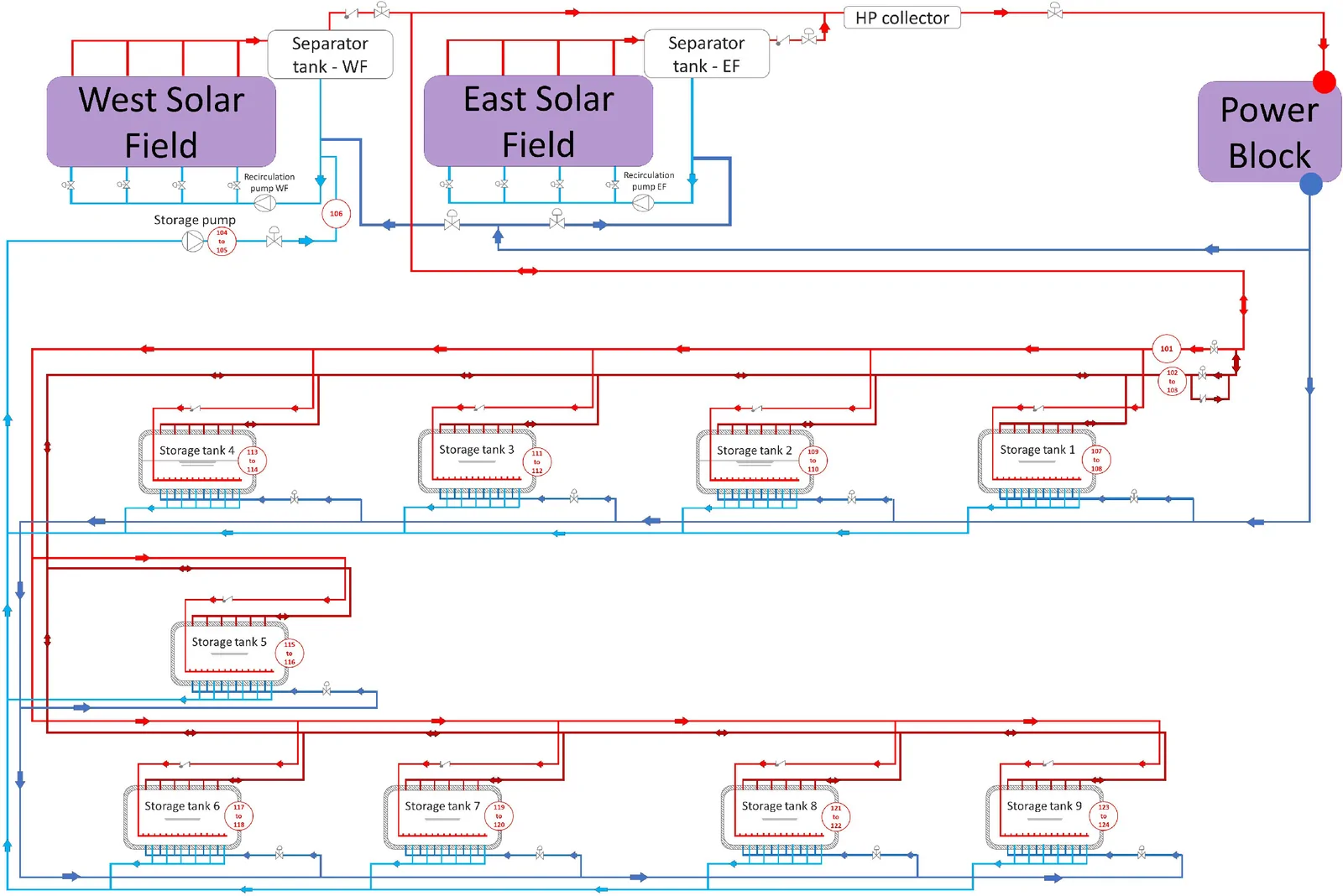
Schematic of the thermal energy storage system instrumentation. Simplified flow diagram of the thermal energy storage system, highlighting the placement of temperature sensors, flow meters, and other monitoring devices within the storage system.

The solar field is oriented northwest/southeast, covering an area of 152,769 m², and is divided into two subfields: the east field (10 solar lines) and the west field (17 solar lines). Each subfield has a separator tank that directs steam to either the power block or storage (for the west field), while water is returned to the solar field.

The power block features a two-stage turbine with a nominal capacity of 10 MWe, equipped with an air-cooled condenser.

The storage system consists of 9 steam accumulators, each with a capacity of 120 m³. The steam is sourced from the west field, and the feedwater comes from the power block.

## 4 Experimental Design, Materials and Methods

### 4.1 Location

The eLLO Concentrated Solar Power plant is located in Llo, Pyrénées-Orientales department, in southern France. Covering an area of approximately 33 hectares (see Fig. 4), the plant features a hilly topography as well as a thalweg and a 63 kV high-tension line (see Fig. 5). Its design is specifically adapted for this site [10] (for an exhaustive treatment, see [11]), which features a hostile environment with cold winters, strong winds, and storms.

**Fig. 4:**
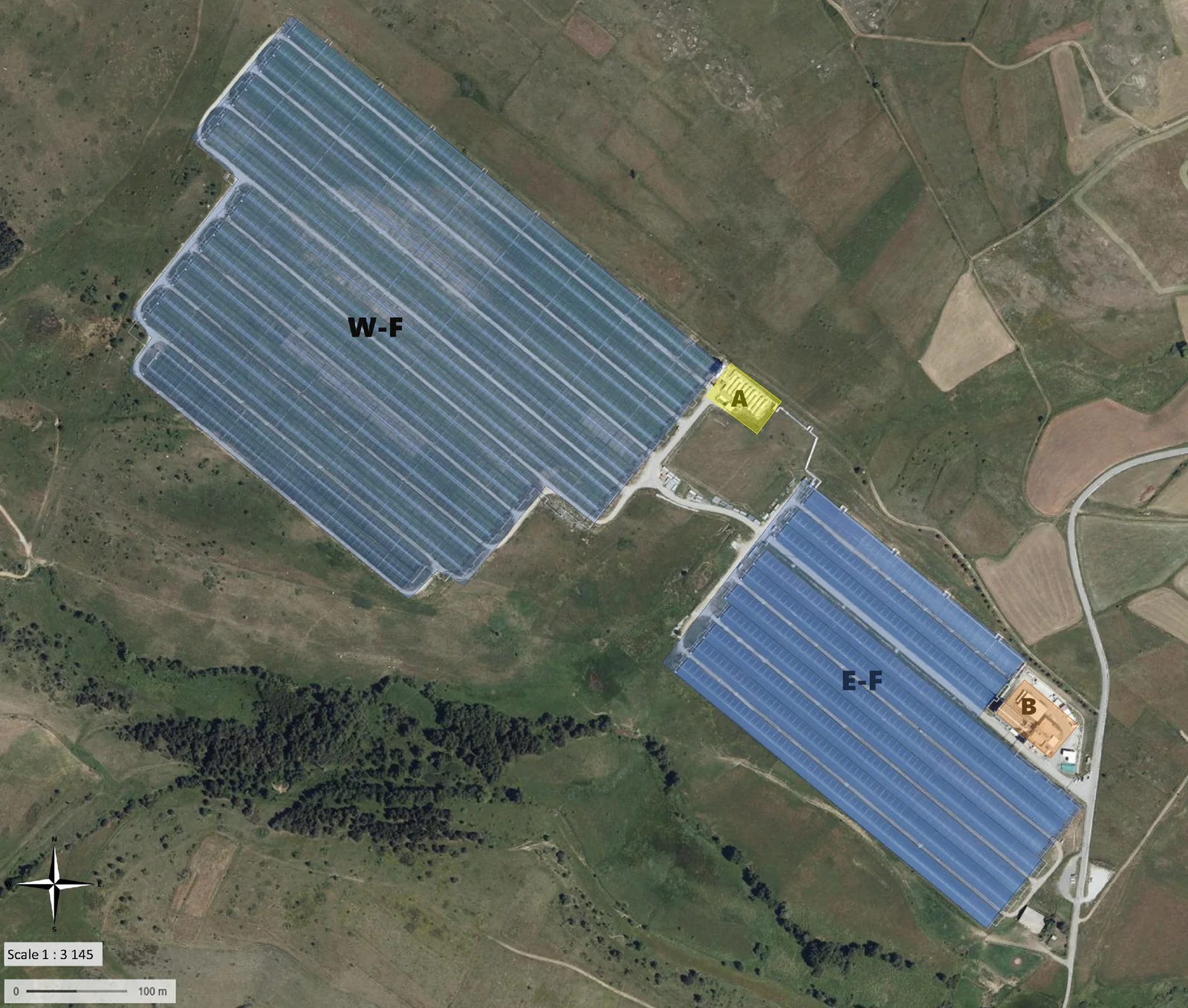
Aerial view of the ELLO solar plant with labeled zones: A (technical West zone with storage system), B (control room and Power Block, including aero condenser and turbine), W-F (West solar Field) and E-F (East solar Field). North is upward. Scale bar: 1:3145.

**Fig. 5:**
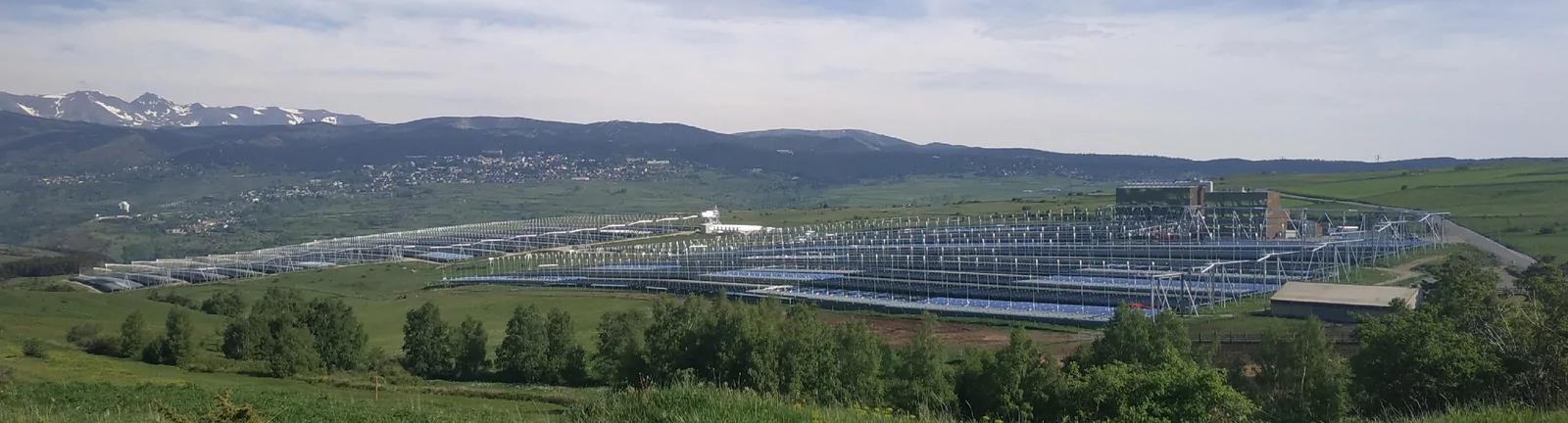
Picture of ELLO’s solar plant, with solar collector lines following the site’s natural topography.

This photograph shows the solar collector lines of the ELLO plant, which follow the site’s natural hilly topography to maximize solar energy capture.

### 4.2 eLLO concentrated solar power plant design

**a. Key parameters.**

The key parameters of the installation are detailed in Table 2.

**Table 2:** Main technical specifications of the system.

Heat Transfer Fluid	Water / Steam
**Nominal Turbine Power**	10 MWe
**Nominal steam conditions at the HP collector**	70 barg, 286 °C
**Storage capacity**	4 h
**Equivalent electrical energy**	40 MWh_e
**Average cycle efficiency**	0.25
**Stored thermal energy**	160 MWh_th
**Total mirror area**	152 769 m²
**Orientation**	Northwest / Southeast

**b. Solar Field.**

The solar field is oriented North-West/South-East due to the specific topography of the location, and divided into two subfields, with the thalweg and high-tension line being the dividing line between both solar fields. Altogether, there are 27 solar lines, 10 in the East solar field, and 17 in the West solar field. Each solar line is formed of 4 to 8 modules associated in series: the East field comprises a total of 54 modules, covering an area of 48,526.6 m², while the West field consists of 116 modules, totaling 104,242.4 m². One module is 67 m x 14 m, and consists of 140 primary reflectors, arranged in 10 longitudinal and 14 transversal spans. Each module is divided into two heliostats with 7 transversal spans, each equipped with its own sun tracking system, including an actuator, a transmission beam and a position encoder, allowing precise adjustment of the mirrors throughout the day.

The fluid exits the solar field at a nominal outlet temperature of 286 °C. The system is designed to operate at a working pressure of 70 barg.

*i. Primary reflector and solar receiver.*

Each primary reflector at the eLLO plant consists of four mirrors bonded under stress to the top plate of a supporting frame, creating a slightly curved reflective surface of about 6.42 m², which enables better concentration compared to flat reflectors.

Key parameters, such as the spacing between rows and primary reflectors, their dimensions, and shape, have been thoroughly investigated and optimized through existing research [12,13]. Moreover, the mirrors used in primary reflectors undergo specialized optical treatments: they are designed to maximize spectral reflectivity (0.94) while minimizing spectral absorptivity.

*ii. Solar receiver.*

The solar receiver is an assembly of components (see Fig. 6) positioned 8.5 m above the mirror’s axis. Its primary function is to intercept concentrated solar radiation and transfer thermal energy to the heat transfer fluid (water/steam).

**Fig. 6:**
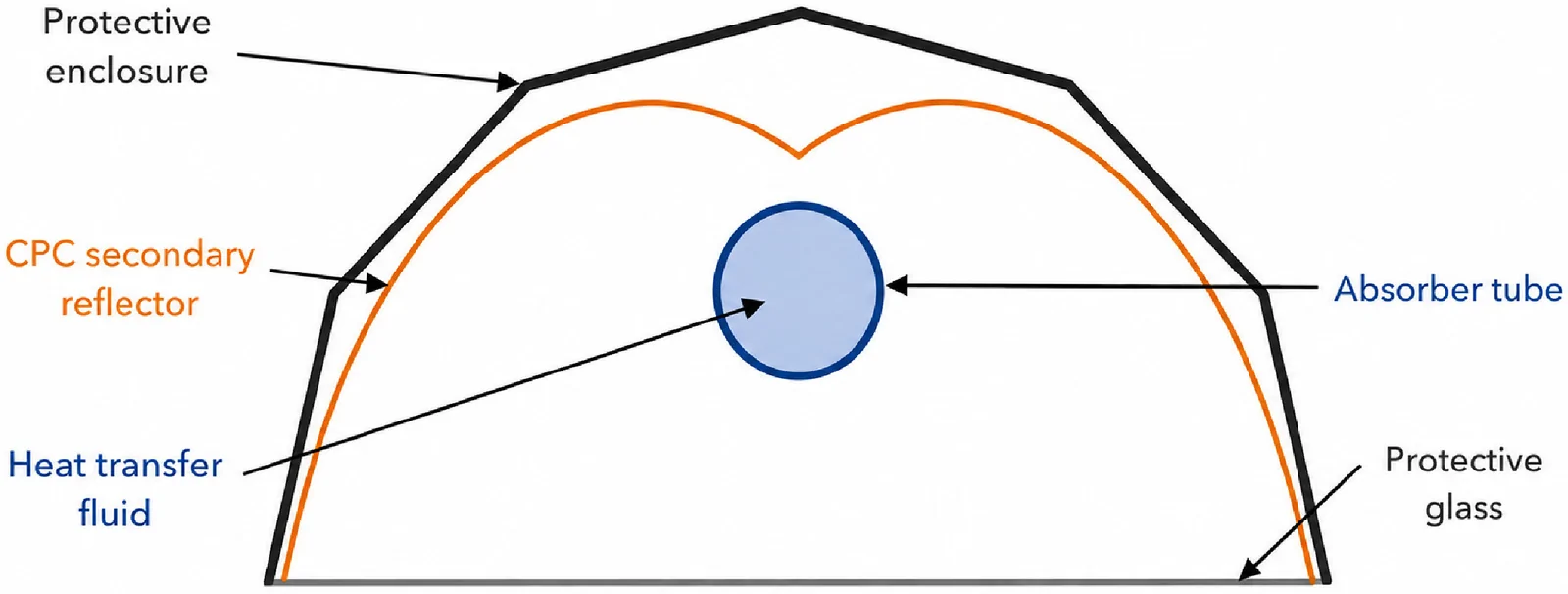
Schematic of a single-tube receiver equipped with a CPC-type reflector. This schematic illustrates the design of a single-tube receiver with a Compound Parabolic Concentrator (CPC)-type reflector.

The solar receiver consists of a 1.4301 (AISI 304) stainless steel absorber tube with selective coating, which converts the radiative solar energy into thermal energy transferred to the heat transfer fluid. It also features a Compound Parabolic Concentrator (CPC-type) secondary reflector, which redirects incoming solar radiation into the absorber tube in order to maximize the collection of diffuse and misaligned solar rays. This overall system is protected by an enclosure and a protective glass in order to minimize thermal losses. In this configuration, the absorber tube is not surrounded by vacuum and the cavity is not insulated.

All surfaces involved in solar concentration are optically treated within the solar radiation wavelength range to minimize optical losses in the system (these optical treatments must withstand mechanical and thermal stresses over time):

• Secondary reflectors are designed to maximize spectral reflectivity (0.89) while minimizing spectral absorptivity.
• Protective glass is designed for high spectral transmittance (ranging from 0 to 0.952 depending on the angle of incidence), and both low spectral reflectivity (ranging from 0 to 0.524 depending on the angle of incidence) and absorptivity. Furthermore, the material used is transparent to solar radiation, but reflective to infrared radiation emitted by the absorber tube (creates a greenhouse effect inside the receiver).
• The absorber tube has a selective coating that maximizes the spectral absorptivity (0.945) in the solar wavelength range, while minimizing spectral emissivity (0.14) in the infrared wavelength range emitted by the absorber.

*iii. Solar tracking system.*

The solar tracking system orients the primary reflectors to effectively concentrate solar radiation onto the receiver, based on the sun’s position. In the linear concentrating system, such as linear Fresnel modules, tracking is performed following a single axis, aligned parallel to the receiver. The rows of primary reflectors are mechanically linked along the rotational axis. The optimal angle for each reflector row is calculated based on the sun’s position (computed in real time by an automated system using specialized algorithms [[14], [15], [16]]) and the row’s relative location to the target (solar receiver).

**c. Direct steam generation (DSG).**

*i. DSG in linear concentrating systems.*

Unlike systems that rely on thermal oils or molten salts as heat transfer fluids, DSG uses water/steam directly within the absorber tubes, simplifying the thermal cycle and reducing operational costs. It offers several advantages, such as eliminating the need for expensive heat transfer fluids and separate steam generator, thus reducing capital and operational costs and exploiting a non-toxic and non-flammable environmentally friendly working fluid.

However, DSG also presents challenges, such as two-phase heat transfer within the absorber tube, which complicates thermal management, but also less efficient internal convection in the gas phase, reducing overall heat transfer performance. Additionally, large-scale thermal storage remains difficult to achieve due to the high volume of steam required and the technical constraints of storing it efficiently.

*ii. Flow configuration.*

The solar fields flow configuration relies on a recirculation architecture, including a steam tank and pumps, to ensure operational continuity and safety in case of a malfunction. The liquid-vapor mixture produced in the solar field, which should reach 80% steam quality, is separated within the steam tank, with the steam routed either to the power block through a superheater, or to the thermal storage system, depending on the pressure differential and the operational strategy, while the liquid water is pumped back into the solar lines by the recirculation pump (see Fig. 7).

**Fig. 7:**
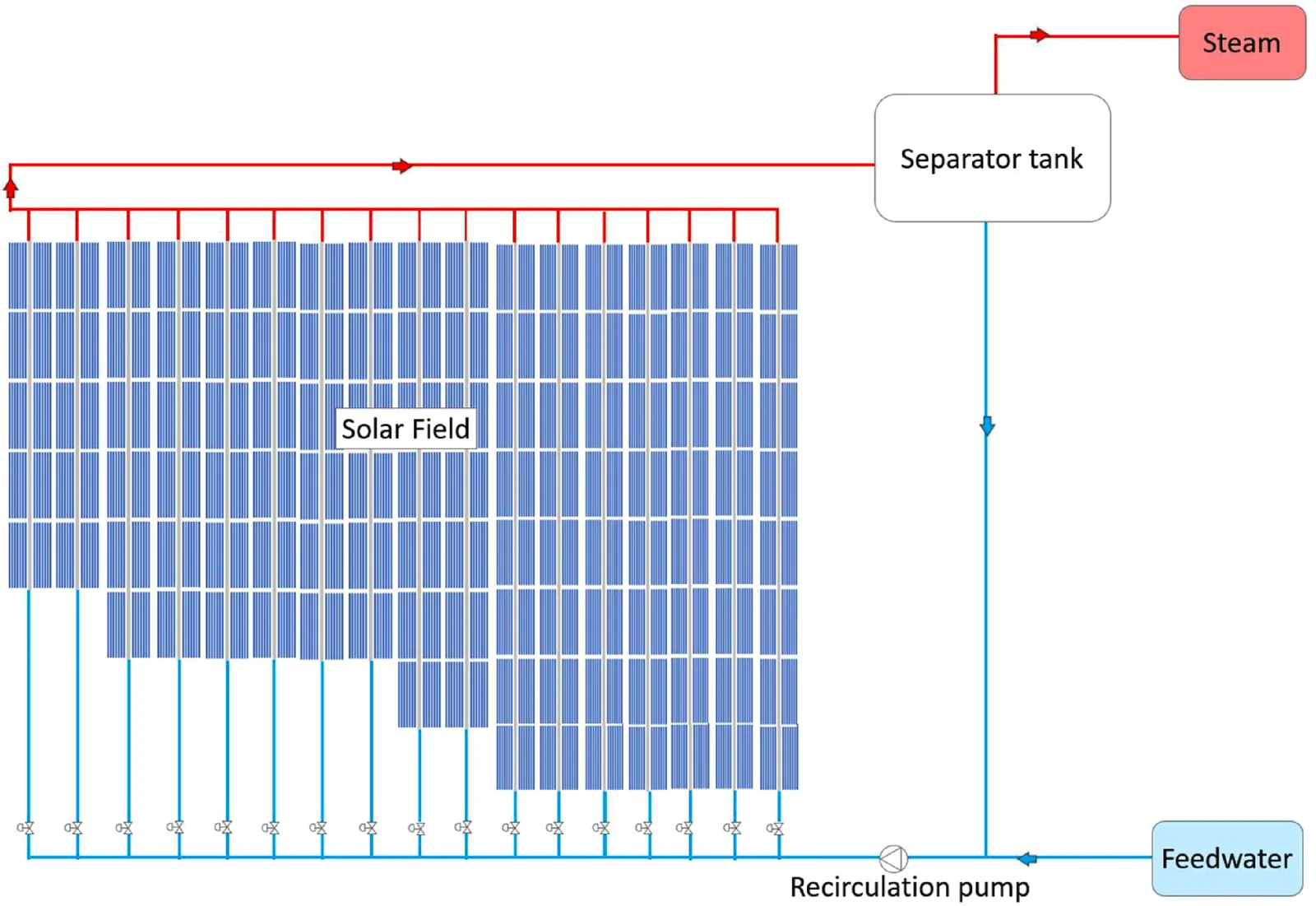
Schematic diagram of the recirculation circuit. This diagram shows a simplified schematic of the recirculation system in the solar field.

**d. Storage.**

While various thermal storage technologies have been explored for CST plants with DSG, steam accumulators stand out due to their rapid-response times and high discharge rates, making them ideal for mitigating the intermittency of solar resources. Steam accumulators store sensible energy in pressurized water: during periods of excess steam production, surplus steam is injected into the liquid phase of the accumulator (see Fig. 8). As the steam injected condenses, it heats the pressurized water to saturation temperature. To maintain the operating pressure, the accumulator’s pressure is controlled via steam injection or extraction, and the water level is regulated by adding or removing liquid. During periods of insufficient steam production, the steam accumulator’s pressure drops, causing flash evaporation of the stored pressurized water, thus releasing saturated steam.

**Fig. 8:**
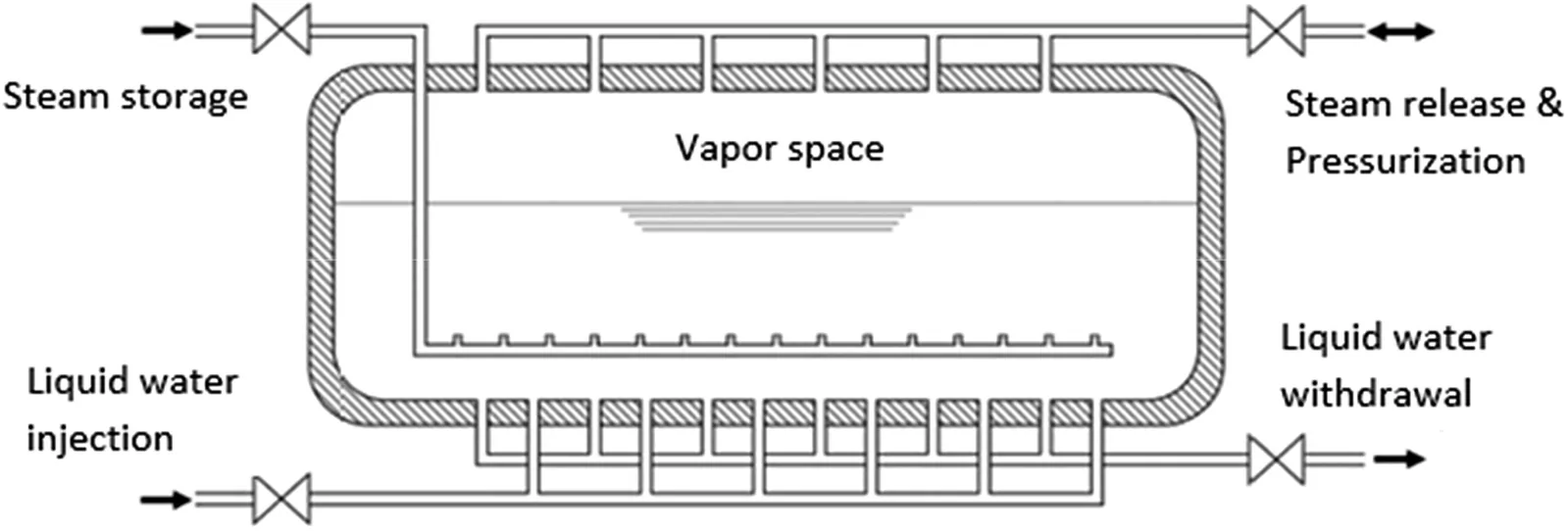
Steam accumulator. This schematic shows a simplified schematic of the steam accumulator showing the inlet and outlet connections for water and steam flow.

This plant’s storage system uses 9 steam accumulators of 120 m³ each, arranged in parallel. This type of storage allows storing surplus steam, with a total capacity of approximately 4 h of full-power operation. The operational strategy depends on the weather: on cloudy days, the storage serves as a buffer, and on sunny days, it acts as isolated storage, used once the solar lines can no longer produce steam.

**e. Power Block.**

The power block operates using a Hirn thermodynamic cycle, a variant of the Rankine cycle. It includes a thermodynamic superheater, a turbo-generator unit, an air-cooled condenser, and several water tanks and heaters.

The turbine consists of two stages, each featuring a dedicated heat exchanger: the superheater (for steam from the solar field) and the reheater (for steam from the first turbine stage). Before entering the turbine, the steam generated in the solar field is superheated. To superheat the steam exiting the first turbine stage, an expansion valve reduces its pressure to 40.5 bar (251 °C), lowering its saturation temperature. This steam is then further heated as it passes through the superheater, which is fed by a portion of the high-pressure saturated steam from the solar field (70 bar, 286 °C). Additionally, the reheater superheats a portion of the steam extracted from the first turbine stage (R1 extraction) to provide the second stage with superheated steam.

The steam expands through the turbine’s multi-stage, driving a 10 MWe turbo-generator unit coupled to an 11 kV alternator to produce electricity. The site self-consumption can be determined by taking the net power production, removing the electricity sent to the grid, and adding the electricity drawn from the grid, using the data provided in the dataset. This gives the actual electricity used on-site.

Condensate is then cooled in the condenser and recirculated through feedwater heaters and pumps, completing the closed cycle. The cycle includes extractions at intermediate turbine stages, used to heat the condensate and facilitate the removal of non-condensable gases.

### 4.3 Acquisition procedures

The dataset was collected from the eLLO concentrated solar power plant, instrumented with multiple sensors, distributed across the solar field, storage system, and power block. The acquisition system is centralized, with all sensors connected to a supervisory data acquisition system, ensuring synchronized logging and remote monitoring. All sensor data are logged by a single, centralized datalogger equipped with a GPS-synchronized clock, ensuring uniform timestamp alignment across the entire site. The datalogger records data at a 1-second resolution, while transmitters apply a 3-second rolling average (damping) to smooth signals for control loops. As such, no inter-logger synchronization is required, and cross-variable timestamp alignment is inherently trustworthy.

**a. Sensors and Instrumentation.**

The sensors and transmitters used in this study are grouped by the type of process variable they measure or transmit, including temperature, pressure, flow, heliostat focusing, meteorological data, tank levels, and electrical power.

Temperature measurements are performed using the Rosemount 148 Temperature Transmitter (model 148HNNAN0Q4XA), a versatile device designed to accept multiple sensors inputs to improve measurement accuracy (with an uncertainty of ±0.3 °C). The 148 series features a 4–20 mA output signal and ambient temperature compensation to ensure reliable performance in diverse environments [17]. In this setup, the transmitter is linked to different temperature sensors (e.g.: 0065J05F0110N0405A1XA) and thermowells (e.g.: EB486D00170100K5N01X) in order to adapt specific conditions such as temperature range and pressure.

For pressure monitoring, Rosemount 2088 Gauge and Absolute Pressure Transmitters (models 2088G3S22A2D4B4Q4Q8S5 and 2088G4S22A2S1D4B4Q4Q8) are used. These transmitters use advanced piezoresistive sensor technology, providing a total accuracy of ±0.075% of span, including combined errors from reference accuracy, ambient temperature effects, and line pressure effects [18]. In this setup, the 2088G3S22A2D4B4Q4Q8S5 is used in most locations, while the 2088G4S22A2S1D4B4Q4Q8 is specifically installed in the storage tank system and the west steam tank.

Flow measurements are conducted with the Rosemount 2051CD Coplanar Pressure Transmitter (model 2051CDCD3A02A1BS5D4V5Q4Q8), part of the 2051 series. It is engineered with patented coplanar technology, enabling direct mounting for flow measurements and providing high accuracy (reference accuracy of ±0.065% of span) and flexibility for a wide range of conditions [19]. The 2051CD is suited for differential pressure flow applications.

Meteorological data are collected using a suite of sensors strategically placed across the plant. It includes measuring Direct Normal Irradiance (DNI), wind speed (0° means south to north, 90° means west to east), ambient temperature and wind directions at various locations. For DNI measurements, the plant uses the following pyrheliometers and sun trackers:

• East DNI is measured using a Kipp & Zonen SHP1 pyrheliometer mounted on a Kipp & Zonen SOLYS2 sun tracker.
• West DNI (output L11) is monitored with a Kipp & Zonen PH1-V pyrheliometer paired with a Kipp & Zonen RaZON+ sun tracker.
• West DNI 2 (input L27) is captured by a Kipp & Zonen SHP1 pyrheliometer on a Kipp & Zonen SOLYS2 sun tracker.

The Global Horizontal Irradiance (GHI) is measured using a Kipp & Zonen CMP11 pyranometer. Finally, wind data is collected using anemometers and wind vanes from Young Wind Monitor 05,103–45.

The system is also equipped with other types of sensors to control and optimize plant performance. Electrical power output and input are tracked in real-time to assess energy production efficiency and grid interaction. Finally, fluid levels in tanks and tanks are tracked using dedicated sensors.

Each sensor is assigned a unique ID, mapped to its location and variable in a metadata file provided with the dataset. The combined data from these diverse monitoring systems offer a complete overview of the plant’s operations.

**b. Measurement Protocol.**

Data were acquired under the plant’s normal operational conditions of the CSP plant, including solar field operation, power block startup/shutdown, and storage charging/discharging.

The dataset was collected over two distinct periods:

• Year 2025, with a 1-hour time step for continuous monitoring.
• July 16, 2025, with a 10-second time step for high-resolution analysis of a best-case day with high DNI and no cloud cover.

For the identification of usable periods, a strict criterion was enforced: time steps containing any NaN values were excluded from the analysis to determine the usability ratio. This resulted in 338 usable days for the year 2025. However, the published dataset retains all time steps, including those with NaN values, to provide a complete and continuous record of the entire year (see Fig. 9).

**Fig. 9:**
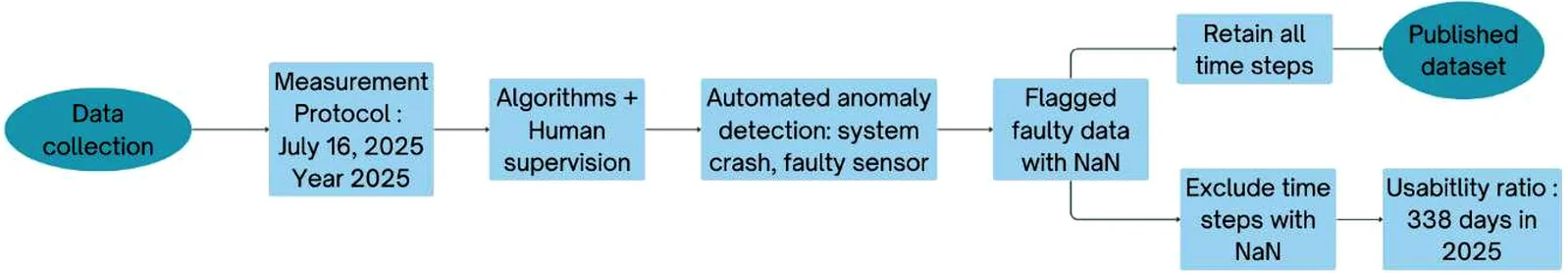
Acquisition data process flowchart. This flowchart shows the process that was used to collect the data.

The anomaly detection process relied on automated flagging performed by the plant’s acquisition system. Only values already identified as NaN (due to system crashes or missing measurements) were retained in their original form. No manual verification or modifications were applied, ensuring that all measurement, including potentially erroneous values, were preserved to maintain internal consistency. Users are advised to assess data coherence for their specific analysis periods, as physically implausible values, such as −15 °C for the “West-field recirculation pump discharge water temperature” parameter, remain unchanged in the dataset. Additionally, small negative values, such as those observed in “Global Horizontal Irradiance (GHI)”, may represent sensor noise and could be treated as zero for analytical purposes, if deemed appropriate.

### 4.4 Operating strategy

The operating conditions reflect the standard daily plant operation. The primary goal is to supply electricity to the grid, with a nominal electrical power target of 9.9MWe. Once this objective is met, the remaining thermal energy will be used to charge the thermal storage system.

All operating setpoints are determined by algorithms, but under continuous operator supervision to ensure safe and reliable plant operation.

The initial phase of an operating day would be the recirculation within the solar field. The fluid will be circulated through the solar field in order to progressively heat the system until the temperature required for power generation is reached.

On-site self-electricity consumption is preferred whenever possible.

The mass flow rate in each solar line is individually regulated as a function of day, time and number of modules. This regulation is achieved through valve opening control to maintain a target steam quality of 0.8 at the outlet and to preserve constant mass in the separator tank of each solar field. The steam quality at the solar field outlet cannot be directly measured and is therefore not included in the published dataset. However, it can be estimated as the ratio between the steam flow from the separator tank and the recirculation flow entering the solar field, using the published variables.

When the thermal storage system is fully charged and the turbine is operating at nominal load, solar modules will be defocused to limit steam production and to ensure stable operating conditions.

## Limitations

The dataset’s main limitation relates to both data availability and data quality. Due to a bug in the central supervision system, some data were not recorded, and entire time periods are missing from the dataset. Additionally, measurement noise and potential issues arising from defective sensors further affect reliability.

Moreover, the dataset lacks critical maintenance records, such as the dates of mirror cleaning operations, details about missing protective glass on the receptor, or documented damage to some mirrors. These gaps introduce uncertainties in interpreting long-term trends and may impact the accuracy of analyses dependent on continuous or high-fidelity data. These limitations have to be taken into account when interpreting these “real world” data.

## Ethics Statement

The authors confirm that they have read and followed the ethical requirements for publication in Data in Brief.

Furthermore, we declare that the current work does not involve human subjects, animal experiments, or any data collected from social media platforms.

The authors confirm that eLLO Power Plant, the operator of the 10 MWe Concentrated Solar Power (CSP) facility in Llo, France, has explicitly consented to the public release of this dataset. This consent was granted by Fabien Roget, production manager at eLLO, who reviewed and approved the dataset and the publication prior to submission.

The data is published under the Etalab Open License 2.0, allowing for open access and reuse.

## CRediT Author Statement

**Valentine Grange:** Data curation, Writing - Original Draft. **Sylvain Rodat**: Writing - Review & Editing, Supervision. **Quentin Falcoz:** Writing - Review & Editing, Supervision. **Valéry Vuillerme:** Writing - Review & Editing, Supervision. **Fabien Roget**: Resources, Supervision

## Data Availability

Recherche Data GouvAnnual operational data for the 10 MWe Ello Fresnel concentrated solar power plant (Original data).
